# Risk factors associated to neural tube defects among mothers who gave birth in North Shoa Zone Hospitals, Amhara Region, Ethiopia 2020: Case control study

**DOI:** 10.1371/journal.pone.0250719

**Published:** 2021-04-26

**Authors:** Aynalem Gashaw, Sisay Shine, Osman Yimer, Melat Wodaje

**Affiliations:** 1 Debre Berhan Town Health Office, North Shoa Zone, Amhara Region, Ethiopia; 2 Public Health Department, College of Health Science, Debre Berhan University, Debre Berhan, Ethiopia; 3 Midwifery Department, College of Health Science, Debre Berhan University, Debre Berhan, Ethiopia; 4 Epidemiology Department, Institute of Health Science, Jimma University, Jimma, Ethiopia; University of Mississippi Medical Center, UNITED STATES

## Abstract

**Introduction:**

Neural tube defects affect the brain and the spinal cord of the developing embryo. The defects occur due to incomplete or failure of closure of the neural tube. The condition eventually causes death and lifelong disability. Worldwide, more than 300,000 babies are born with neural tube defects each year. The highest burden is in low- and middle-income countries. Therefore, this study aims to identify the risk factors associated with neural tube defects among mothers who gave birth in North Shoa Zone Hospitals.

**Methods:**

A hospital based unmatched case-control study was conducted among 243 (81 cases and 162 controls) study participants in North Shoa Zone Hospitals. The hospitals were selected using simple random sampling and all cases and randomly selected controls in the selected hospitals were included in the study. The data were collected by using pre-tested structured questionnaire.

**Results:**

Different factors were identified to have association with neural tube defect. Family annual cash income less than 24000ETB (AOR: 3.73, 95%CI: 1.35, 10.26), history of still birth (AOR: 3.63, 95%CI: 1.03, 12.2), history of abortion (AOR: 6.15, 95%CI: 2.63, 18.56), preconception tea use (AOR: 2.36, 95%CI: 1.15, 4.86) and pesticides/chemical exposure (AOR: 5.34, 95%CI: 1.77, 16.05) were positively associated factors. In contrast, preconception care (AOR: 0.14, 95%CI: 0.05, 0.39) and taking iron/folic acid/multivitamin during the current pregnancy (AOR: 0.16, 95%CI: 0.07, 0.33) showed a protective effect.

**Conclusion:**

Family annual income less than 24000ETB, history of still birth, history of abortion, preconception tea uses and pesticides/chemical exposure were associated factors of neural tube defects. Preconception counseling and screening should be recommended for women who plan for pregnancy.

## Introduction

Neural tube defects(NTDs) are structural defect of the central nervous system that affect the brain and spinal cord of the developing embryo [1, 2]. NTDs occur due to incomplete or failure of closure of the neural tube [3]. The defects develop during early weeks of intrauterine development [4]. Failure of closure of the neural tube during development results in spina bifida, anencephaly and encephalocele. These are serious and the leading causes of infant and child mortality, morbidity and lifelong disabilities [5, 6].

Advance in medical technology increases survival rates of babies born with birth defects. But, this requires significant financial resources for the long-term care of individuals with disabilities. There is also significant burden on healthcare systems and caregiver time. It also causes great social and emotional costs for children with NTDs and their families [7].

NTDs affect not only the life of the child and their families but also the community as well. Anencephaly is fatal, either in utero or immediately after birth and child with spina bifida face limited brain growth. Bladder and bowel dysfunction and paralysis of the lower limbs are common because of lack of early medical and surgical treatment. The problem raises great economic, educational and emotional issues, and require special healthcare and social services to improve the quality of life and social involvement [6, 8].

Worldwide, more than 300,000 babies are born with NTDs each year. The highest burden is in low and middle income countries specifically Africa [9, 10].

Any women of child-bearing age are at risk of having a pregnancy affected by NTDs. It is impossible to predict which women will have a pregnancy affected by NTDs [11]. The exact cause of NTDs is not known. Ninety-five percent of women with NTDs affected pregnancy have no personal or family history of NTDs. However, some genetic, environmental and nutritional factors are considered to contribute for the occurrence of NTDs [12–14]. Micronutrient insufficiency, maternal lifestyle like intake of recreational drugs, smoking and consumption of alcohol during pregnancy were significantly associated with NTDs [2, 15, 16]. In addition, maternal conditions like obesity and age have contribution for the occurrence of NTDs. Infectious diseases and environmental exposures to different pollutants also have a role [13, 14, 17, 18]. Moreover, personal or family history of NTDs, use of anti-seizure and anti-diabetes medications increase the risk of having NTDs affected offspring [11].

However, in Africa, particular in Ethiopia, there is limited data, and also there is variation in risk factors by geographic location. Information about NTDs in North Shoa Zone, the study area of this study, is rare. Therefore, this study aimed to identify risk factors for Neural Tube Defects in North Shoa Zone.

## Methods

### Study design, setting and population

Hospital based unmatched case control study design was conducted from October 2019 to April 2020 among mothers who gave birth in North Shoa Zone hospitals, Amhara Region, Ethiopia. Women of age 15–49 are estimated to be 20.23% of the total population and there are 9 public hospitals [19]. All mothers who gave birth in the hospitals of North Shoa Zone were the source population for this study.

### Sample size and sampling strategies

The sample size was calculated considering unmatched case control formula for annual family cash income less than 1300 USD, proportion among controls 20.07%, proportion among cases 38.6% and with odds ratio of 2.5 [20], 5% margin of error, 95% confidence level and 80% power. Considering 5% non-response rate and case to control ratio of 1:2, the final sample size was 243 with 81 cases and 162 controls.

Among the nine government hospitals five, Minjar Shenkora, Deneba, Merhabetie Enat, Mahel Meda and Debre Brehan hospitals were selected randomly. Samples were allocated for each hospital based on the last one-year estimation of average birth and case flow. In the sample selection all consecutive cases were included and controls were selected by simple random sampling method from the same institutions.

### Operational definition

#### Neural tube defects (NTDs)

Cases of anencephaly, spina bifida and Encephalocele [5].

#### NTDs cases

Mothers, who gave birth for a child with any type of NTDs, irrespective of gestational age and live birth.

#### NTDs controls

Mothers, who gave birth for a child without any type of NTDs and other congenital anomaly on their babies.

### Data collection methods and instrument

Structured questionnaire was developed by reviewing different literatures of similar studies and WHO guideline [16, 20, 21]. The questionnaire contains items on socio-demographic characteristics, reproductive history, medical history, behavioral, environmental characteristics and the status of NTDs. To maintain the consistency English version questionnaire was translated into Amharic and retranslate back to English. The questionnaire was pre-tested on 5% of sample size. Cases were ascertained by senior obstetrics and gynecology specialists, radiologists and integrated emergency surgical officers by gross visual examination and by using ultrasonography. Diagnosis was confirmed after expulsion of medically terminated abortion and delivery of the fetus by the clinicians.

### Statistical analysis

Data were entered to Epi-Info 7.0.9.7 version computer software package and exported to SPSS window version 20 for data management and analysis. Editing, cleaning, and checking for completeness and consistency was done. Descriptive analysis was done to summarise the characteristics of study participants. Both bivariate and multivariate logistic regression analysis was done to identify the risk factors associated withNTDs. Variables with P value < 0.25 during the bivariate analysis were included in the multivaribabe logistic regression model to control the effect of confounding variables and identify statistically significant associated factors. Adjusted odds ratio with 95% confidence interval was calculated and variables with P-value less than 0.05 were considered as statistically significant risk factors to NTDs. Finally, data was presented using graphs, tables and statements.

### Ethical approval and consent to participate

Ethical clearance was obtained from Debre Berhan University college of health sciences research ethics review committee. Informed written consent was obtained from each study participant prior to data collection.

## Results

### Socio-demographic characteristics of the study participants

A total of 243 participants with 81 cases and 162 controls were included with 100% response rate. Among the total 81 NTDs cases 44 (54.3%) were anencephaly, 31 (38.3%) spina bifida and 6(7.4%) encephalocele. The mean age of the mothers was found to be 26.77 the SD of ±5.24 years old. About 92.6% cases and 96.9% control married. Regarding occupation, more than 40% of cases and 37% of controls were housewife (Table 1).

**Table 1:** Socio-demographic characteristics of mothers who gave birth in North Shoa Zone Hospitals, Amhara Region, Ethiopia, 2020.

Variables	Cases: n (%)	Controls: n (%)	Total: n (%)
Maternal age			
<20	8(9.9)	20 (12.3)	28 (11.5)
21–25	29 (35.8)	52 (32.1)	81 (33.3)
26–30	26 (32.1)	61 (37.7)	87 (35.8)
31–35	11 (13.6)	21 (13)	32 (13.2)
≥35	7 (8.6)	8 (4.9)	15 (6.2)
Marital status			
Single	6 (7.4)	5 (3.1)	11 (4.5)
Married	75 (92.6)	157 (96.9)	232 (95.5)
Occupational status			
Housewife	33 (40.7)	60 (37)	93 (38.3)
Farmer	13 (16)	24 (14.8)	37 (15.2)
Merchant	15 (18.5)	27 (16.7)	42 (17.3)
Office worker	20 (24.7)	51 (31.5)	71 (29.2)
Residence			
Urban	46 (56.8)	118 (72.8)	164 (67.5)
Rural	35 (43.2)	44 (27.2)	79 (32.5)
Maternal educational status			
No formal education	19(23.5)	28 (17.3)	47 (19.3)
Primary school	14 (17.3)	21 (13)	35 (14.4)
Secondary school	28 (34.6)	69 (42.6)	97 (39.9)
Above secondary	20 (24.7)	44 (27.2)	64 (26.4)
Paternal educational status			
No formal education	24 (29.6)	24 (14.8)	48 (19.7)
Primary school	7 (8.6)	18 (11.1)	25 (10.3)
Secondary school	29 (35.8)	66 (40.8)	95 (39.1)
Above secondary	21 (25.9)	54 (33.3)	75 (30.9)
Family annual cash income			
< 24000	33 (40.7)	37 (22.8)	70 (28.8)
24001–36000	22 (27.2)	38 (23.5)	60 (24.7)
36001–60000	17 (21)	39 (24.1)	56 (23)
≥60000	19 (11.1)	48 (29.6)	57 (23.5)

### Reproductive history of the study participant

Among the study participants, 36(44.4%) of case and 70(43.2%) of controls had their first pregnancy. About 17(21%) of cases and 10(6.2%) of controls had aborted. Related to current pregnancy, 18(22.2%) of cases and 72(44.4%) of controls-initiated ANC follow up in their first trimester. The pregnancy for more than 87% of controls and 64.2% of cases was planned (Table 2).

**Table 2:** Reproductive history of mothers who gave birth in North Shoa Zone Hospitals, Amhara Region, Ethiopia, 2020.

Variables	Cases: n (%)	Controls: n (%)	Total: n (%)
Gravidity			
First	36 (44.4)	70 (43.2)	106 (43.6)
Second	15 (18.5)	35 (21.6)	50 (20.6)
Third and above	30 (37)	57 (35.2)	87 (35.8)
History of still birth			
Yes	12 (14.8)	5 (3.1)	17 (7)
No	69 (85.2)	157 (96.9)	226 (93)
History of abortion			
Yes	17 (21)	10 (6.2)	27 (11.1)
No	64 (79)	152 (93.8)	216 (88.9)
Family history of abortion			
Yes	6 (7.4)	1 (0.6)	7 (2.9)
No	75 (92.6)	161 (99.4)	236 (97.1)
Last child breast feeding time			
No previous child	48 (59.3)	81 (50)	129 (53.1)
≤2 years	28 (34.6)	69 (42.6)	97 (39.9)
>2 year	5 (6.2)	12 (7.4)	17 (7)
ANC initiation time			
Not have	20 (24.7)	3 (1.9)	23 (9.5)
1st trimester	18 (22.2)	72 (44.4)	90 (37)
2nd trimester	35 (43.2)	75 (46.3)	110 (45.3)
3rd trimester	8 (9.9)	12 (7.4)	20 (8.2)
Planned pregnancy			
Yes	52 (64.2)	141 (87)	193 (79.4)
No	29 (35.8)	21 (13)	50 (20.6)

## Medical history of the study participants

About 41.4% of controls and 8.6% of cases received preconception care. Among the total respondent, 16% of cases and 4.3% of controls had history of NTDs before the current pregnancy. Only few of the cases and controls took iron/ folic acid/multivitamin before conception (Table 3).

**Table 3:** Medical history of mothers who gave birth in North Shoa Zone Hospitals, Amhara Region, Ethiopia, 2020.

Variables	Cases: n (%)	Controls: n (%)	Total: n (%)
Preconception care			
Yes	7 (8.6)	67 (41.4)	74 (30.5)
No	74 (91.4)	95 (58.6)	169 (69.5)
Any chronic illness before conception			
Yes	11 (13.6)	3(1.9)	14 (5.8)
No	70 (86.4)	159(98.1)	229 (94.2)
Preconception illness (fever)			
Yes	15 (18.5)	8 (4.9)	23 (9.5)
No	66 (81.5)	154 (95.1)	220 (90.5)
Preconception sever vomiting			
Yes	5 (6.2)	9 (5.6)	14 (5.8)
No	76 (93.8)	153 (94.4)	229 (94.2)
Take any drug during preconception			
Yes	14 (17.3)	6 (3.7)	20 (8.2)
No	67 (82.7)	156 (96.3)	223 (91.8)
Previous history of NTDs affected pregnancy			
Yes			
No		7 (4.3)	20 (8.2)
Family history of NTDs affected pregnancy	13 (16)	155 (95.7)	223 (91.8)
Yes	68 (84)		
No		1 (0.6)	11 (4.5)
Take iron/ folic acid/multivitamin before conception			
Yes	10 (12.3)	161 (99.4)	232 (95.5)
No	71 (87.7)	19 (11.7)	24 (9.9)
Take iron/ folic acid/multivitamin during the current pregnancy			
Yes	5 (6.2)	143 (88.3)	219 (90.1)
No	76 (93.8)	124(76.5)	155 (63.8)
History of nutritional deficiency	31 (38.3)	38 (23.5)	88 (36.2)
Yes	50 (61.7)		
No		6 (3.7)	13 (5.3)
	7 (8.6)	156 (96.3)	23(94.7)
	74 (91.4)		

### Behavioral and environmental characteristics of the study participant

Most of the study participants, 59(72.8%) of cases and 108(66.7%) of controls, used coffee as a source of caffeine during the preconception period. And also, 54(66.7%) of cases and 81(50%) controls used tea during the preconception period. About 15(18.5%) of cases and 11(6.8%) of controls were exposed to pesticides/chemical during the preconception period (Table 4).

**Table 4:** Behavioral and environmental characteristics of mothers who gave birth in North Shoa Zone Hospitals, Amhara Region, Ethiopia, 2020.

Variables	Cases: n (%)	Controls: n (%)	Total: n (%)
Preconception coffee use			
Yes	59 (72.8)	108 (66.7)	167 (68.7)
No	22 (27.2)	54 (33.3)	76 (31.3)
Coffee amount/day(average)			
<3 cup	51 (86.4)	96 (87)	145 (86.8)
≥3 cup	8 (13.6)	14 (13)	22 (13.20
Preconception tea use			
Yes	54 (66.7)	81 (50)	135 (55.6)
No	27 (33.3)	81 (50)	108 (44.4)
Tea amount/day(average)			
<3 cup	51 (94.4)	80 (98.8)	131 (97)
≥3 cup	3 (5.6)	1 (1.2)	4 (3)
Preconception alcohol use			
Yes	18 (22.2)	53 (32.7)	71 (29.2)
No	63 (77.8)	109 (67.3)	172 (70.8)
Passive cigarette smoking			
Yes	7 (8.6)	5 (3.1)	12 (4.9)
No	74 (91.4)	157(96.9)	231 (95.1)
Preconception pesticides/chemical use			
Yes	15 (18.5)	11 (6.8)	26 (10.7)
No	66 (81.5)	151 (93.2)	217 (89.3)
Source of drinking water			
Pipe	62 (76.2)	135 (83.3)	197 (81.1)
Well	9 (11.1)	12 (7.4)	21 (8.6)
Spring	10 (12.3)	15 (9.3)	25 (10.3)
Preconception X-ray exposer			
Yes	3 (3.4)	4 (2.5)	7 (2.9)
No	78 (96.3)	158 (97.5)	236 (97.1)

### Factors associated with NTDs

A bivariate and multivariable logistic regression was conducted to identify factors associated with NTDs. In the bivariate analysis factors like maternal residence, no formal paternal education, family annual cash income less than 24000ETB and 24001-3600ETB, history of still birth, history of abortion, unplanned pregnancy, preconception care, preconception illness(fever), taking any drug during preconception period, history of NTDs, taking iron/ folic acid/multivitamin during the current pregnancy, preconception tea use and preconception pesticides/chemical exposure were identified to be related to NTDs and included to the final multivariable logistic regression.

In multivariable logistic regression, having family annual cash income less than 24000ETB (AOR: 3.73, 95%CI: 1.35, 10.26), history of still birth (AOR: 3.63, 95%CI: 1.03, 12.2), history of abortion (AOR: 6.15, 95%CI: 2.63, 18.56), having preconception care (AOR: 0.14, 95%CI: 0.05, 0.39), taking iron/ folic acid/multivitamin during the current pregnancy (AOR: 0.16, 95%CI: 0.07, 0.33), using tea during preconception period (AOR: 2.36, 95%CI: 1.15, 4.86) and preconception pesticides/chemical exposure (AOR: 5.34, 95%CI: 1.77, 16.05) were identified to be significantly associated factors with NTDs. But, maternal residence, no formal paternal education, unplanned pregnancy, preconception illness (fever), taking any drug during preconception, history of NTDs did not show significant association with NTDs (Table 5).

**Table 5:** Factors associated with NTDs among mothers who gave birth in North Shoa Zone Hospitals; Amhara Region, Ethiopia, 2020.

Variables	Cases: n (%)	Controls: n (%)	COR (95%CI)	AOR (95%CI)	P value
Residence					
Urban	46 (56.8)	118 (72.8)	1	1	
Rural	35 (43.2)	44 (27.2)	2(1.16–3.57)	1.67(0.86–3.25)	0.132
Paternal educational status					
No formal education	24 (29.6)	24 (14.8)	2.57(1.21–5.48)	1.34(0.53–3.37)	0.538
Primary school	7 (8.6)	18 (11.1)	1(0.36–2.74)	0.62(0.21–1.84)	0.389
Secondary school	29 (35.8)	66 (40.8)	1.13(0.58–2.2)	0.76(0.36–1.6)	0.471
Above secondary	21 (25.9)	54 (33.3)	1	1	
Family annual cash income					
< 24000	33(40.7)	37(22.8)	4.75(2–11.15)	3.73(1.35–10.26)	0.011*
24001–36000	22(27.2)	38(23.5)	3.11(1.27–7.47)	2.45(0.84–7.18)	0.102
36001–60000	17(21)	39(24.1)	2.32(0.93–5.78)	1.86(0.21–1.84)	0.273
≥60000	19(11.1)	48(29.6)	1	1	
History of still birth					
Yes	12 (14.8)	5 (3.1)	5.46(1.85–16.0)	3.63(1.03–12.2)	0.045*
No	69 (85.2)	157 (96.9)	1	1	
History of abortion					
Yes	17 (21)	10 (6.2)	4(1.75–9.29)	6.15(2.63–18.57)	0.001*
No	64 (79)	152 (93.8)	1	1	
Planned pregnancy					
Yes	52 (64.2)	141 (87)	1		
No	29 (35.8)	21 (13)	3.75(1.96–7.1)	1.78(0.78–4.03)	0.165
Preconception care					
Yes	7 (8.6)	67 (41.4)	0.13(0.05–0.3)	0.14(0.05–0.39)	0.001*
No	74 (91.4)	95 (58.6)	1	1	
Preconception illness (fever)					
Yes	15 (18.5)	8 (4.9)	4.37(1.76–10.8)	2.29(0.78–6.77)	0.131
No	66 (81.5)	154 (95.1)	1	1	
Take any drug during preconception					
Yes	14 (17.3)	6 (3.7)	5.43(2–14.74)	3.72(0.99–13.78)	0.51
No	67 (82.7)	156 (96.3)	1	1	
History of NTDs					
Yes	13 *(*16)	7 (4.3)	4.23(1.61–11.1)	2.11(0.56–7.85)	0.268
No	67 (84)	155 (95.7)	1	1	
Take iron/ folic acid/multivitamin during the current pregnancy					
Yes	31 (38.3)	124(76.5)	0.19(0.10–0.33)	0.16(0.07–0.33)	0.001*
No	50 (61.7)	38 (23.5)	1	1	
Preconception tea use					
Yes	54 (66.7)	81 (50)	2(1.14–3.48)	2.36(1.15–4.86)	0.019*
No	27 (33.3)	81 (50)	1	1	
Preconception pesticides/chemical use					
Yes	15 (18.5)	11 (6.8)	3.12(1.36–7.15)	5.34(1.77–16.05)	0.003*
No	66 (81.5)	151 (93.2)	1		

* Significant at P<0.05.

## Discussion

This hospital-based case control study was conducted to assess the risk factors of NTDs among mothers who gave birth in North Shoa Zone Hospitals. Having pregnancy in developing countries is to be at risk for potential predisposing factors such as infection, lower socioeconomic and educational status, low environmental protection from exposers and low access to medication [10, 22, 23]. Establishing intervention plan for prevention of birth defects depends on information about the possible risk factors [24].

The result of this study revealed that family annual cash income less than 24000ETB was associated with NTDs. This result is consistent with case control study conducted in Addis Ababa, Ethiopia [20]. But, it is inconsistent with case control study conducted in Bale Zone, Ethiopia [25]. This could be due to productivity difference; more cash crops like coffee are planted in Bale Zone than North Shoa Zone [26]. Low family income could result to under nutrition of the family. Maternal nutritional problem causes so many fetal and child disabilities and developmental problems [27].

In the current study, history of still birth and abortion were identified to be risk factors of NTDs. The result is similar with study conducted in Tigray, Ethiopia. It indicates that there was association between still birth and NTDs [16]. The result is consistent with case control studies done in South Africa and Kashan, Central Iran. There was strong association between abortion and having NTDs [28, 29]. While, in the studies of Bale Zone, Ethiopia and Western Iran, there was no association between abortion and NTDs [24, 25]. This gap could be due to the difference in sample size. A woman who had miscarriages in the past has a higher risk of having a baby with birth defects like neural tube defects. It could be due to the increased risk of recurrence. A study indicates that recurrence risk of NTDs was around 1–3% [29]. Moreover, when the fetus is affected by congenital abnormality like NTDs could end with spontaneous abortion [30].

Mothers who have preconception care were 86% less likely to give birth to babied with NTDs. The possible assumption could be when women getting counseling and screening could prevent communicable and non-communicable disease. It also creates healthy environment before conception to improve maternal and child health outcomes.

This study revealed that mothers who take iron/folic acid/multivitamin during the current pregnancy were 84% less likely to have babies with NTDs. The result is in line with the studies conducted in Bangladesh, Slone Epidemiology Center at Boston, Saudi Arabia and Bale Zone, Ethiopia [25, 31–33]. However, it is inconsistent with a study conducted in Kashan, Central Iran [29]. As shown in different study using folic acid or multivitamins could reduce the risk of NTDs. For instance, inappropriate medication use could reduce its action. Different studies evidenced that daily supplementation of 400 micrograms of folic acid is effective in reducing the occurrence and recurrence of NTDs.

Taking tea during the preconception period was an identified to have association with NTDs. Caffeine is a natural component of coffee, tea, and cocoa. It can cross the placenta during pregnancy and high levels of caffeine consumption during gestation produce teratogen effects resulting in malformations, including NTDs [34]. In addition, using black and green tea can be problematic during pregnancy due to its ability to lower maximum blood concentration of folic acid in humans.

In this study preconception exposure to pesticide/chemical was found to be associated with NTDs. The result is consistent with a case control study done in the University of Iowa [35] Bur different from similar case control study in Tigray, Ethiopia [16]. People use pesticide/chemical to prevent peste, rodents and different insects. However, pesticides compounds or chemicals could be an important source of developmental toxins and teratogen and the nervous system is particularly susceptible to many pesticides and chemicals [36].

With all its important outcomes, this study has also limitations worth to be mentioned. Since it is a hospital-based study, it is difficult to be generalized to the general population. The use of small study samples might affect the generalizability and the model’s ability to show association between risk factors and outcome variables. The exposure status of the study participants was assessed retrospectively and this may be affected by a recall bias. In addition, the estimates of the model might be unstable due to the presence of very small numbers in some categories which results in lack of positivity/sparsity.

## Conclusion and recommendation

The result of this study revealed that family annual cash income less than 24000ETB, history of still birth and abortion, preconception tea use and preconception pesticides/chemical exposer were significantly associated with NTDs. Whereas, preconception care and taking iron/folic acid/multivitamin during the current pregnancy were reduce the risk of NTDs. Therefore, preconception counseling and screening should be done for women who will plan for pregnancy. In addition, this research was depending on self-report, more studies with better exposure assessment are important.

## Supporting information

S1 File English version questionnaire. (DOCX)

S2 File Amharic version questionnaire. (DOCX)

## Abbreviation

ANC: Antenatal Care
AOR: Adjusted Odd Ratio
CI: Confidence Interval
ETB: Ethiopian Birr
NTD: Neural Tube Defect
SD: Standard Deviation
