# Supplementary material for: Risk factors associated to neural tube defects among mothers who gave birth in North Shoa Zone Hospitals, Amhara Region, Ethiopia 2020: Case control study
Source: PLoS One. 2021 Apr 26;16(4):e0250719. doi: 10.1371/journal.pone.0250719 (PMC8075213; doi:10.1371/journal.pone.0250719)
Supplement: S1 File — (DOCX) [file pone.0250719.s001.docx]

English Version Questioner

Information sheet and consent form /English version

Information sheet

Date________ EC _________GC

Introduction

Dear participants!

My name is ____________ and I am MPH student at Debre Berhan University, collage of health Sciences and department of public health. I would like to ask few questions which take _______minutes about determinant factors of neural tube defect (NTDs) among mothers. Your genuine information that you are going to provide will help for prevention and control of NTDs. As participant of this study, you give me consent after you have understood the following information sheet:

**Title of the study**: Determinants of neural tube defects among mothers gave birth in North Shoa Zone hospitals, Amhara regional state, Ethiopia.

**Objective**-The main aim of this study is to assess the determinant factors of neural tube defect (NTDs) among mothers gave birth in North Shoa Zone hospitals.

**Risks**- There is no risk in participating in this research.

**Benefits**-There is no immediate benefit in participating in this study. However, your participation will contribute in improving maternal health care.

**Incentive**-There is no financial or material incentive in participating in this study.

**Confidentiality**- - Any information forwarded will be kept private and your name will not specified.

**Participant rights-** Your participation is entirely voluntary and up to you to decide. There is no penalty if you do not agree to participate. Also you have the right not to answer any questions you do not want to. You may also withdraw from the study at any time. If in the middle you decide to stop filling questions and no longer participate, you can stop without worry. If you have any question, you can ask at any time. If you have additional questions about the study, you can contact with:

**Principal investigator-** Aynalem Gashaw, Email ayni581@gmail.com, phone +251-975259688

Thank you for your cooperation. If you are voluntary to participate in the study we kindly request you to provide your response for the questionnaire in the next page.

**Informed consent**

If you have read this form or it has been read to you in the language you comprehend and understood all conditions stated above. Therefore, would you willing to participate in this study.

Yes_________ No_________

Respondent’s signature__________

Interviewer name_____________

Name of principal investigator Aynalem Gashaw Contact Email ayni581@gmail.com Phone +251-975259688

Ethical and research review board of DBU Phone; ________________

Date of interview ________

Result of interview: 1.Completed 2.Respondent not available 3. Refused 4. Partially completed.

If the respondent is not voluntary, please skip to the next participant.

| Variable | Questions | | Answer | Remark |
| --- | --- | --- | --- | --- |
|  | Does she have NTD on her fetus/baby?  (Don’t ask the woman, see it and fill) | | 1. No 2. Yes |  |
|  | If yes, which type of NTD dose her fetus/baby has?  (Don’t ask the woman, see it and fill) | | 1. Anencephaly 2. Spina bifda   3. Encephalocele |  |
| Part I: Socio-demographic factor | | | |  |
| S.no | |  |  |  |
| 101 | | How old are you? | ________ completed years |  |
| 102 | | Marital status | 1. Single  2. Married |  |
| 103 | | What is your occupation? | 1. Housewife  2. Farmer  3. Merchant  4. Office worker |  |
| 104 | | What is your residence? | 1. Urban 2. Rural |  |
| 105 | | Do you have formal education? | 1. No 2. Yes |  |
| 106 | | If yes Q.No 105 what is your highest educational level? | ____________ |  |
| 107 | | Does your husband have formal education? | 1. No 2. Yes |  |
| 108 | | If yes Q.No 107what is he’s highest educational level? | ____________ |  |
| 109 | | How many kuntal agricultural products did you get per year on average? (Rural dweller) | ____________ kuntal |  |
| 110 | | How much is your family annual income (in birr)?  (urban dweller) | ________in birr |  |
| Part II: Reproductive history | | | | |
| 201 | | How many pregnancies do you have?  (number of total pregnancies) | ________ pregnancy/pregnancies |  |
| 202 | | Do you have history of still birth? | 1. No 2. Yes |  |
| 203 | | Do you have history of abortion? | 1. No 2. Yes |  |
| 204 | | Had history of abortion in your/husband family? | 1. No 2. Yes |  |
| 205 | | For how long you breast feed your last child? | __________months |  |
| 206 | | When did you start ANC visit for the current pregnancy | _________ completed weeks |  |
| 207 | | Was the current pregnancy planned? | 1. No 2. Yes |  |
| Part III Medical history related factors | | | | |
| 301 | | Did you get advice or treatment for lifestyle modification before this pregnancy?( screened for any disease and get treatment, take folic acid, take vaccine, get counseling, modify diet, cessation of alcohol and cigarette smoking, stop taking of illegal drugs, plan pregnancy and create healthy environment) | 1. No 2. Yes |  |
| 302 | | Do you have any chronic illness told by a doctor? (Chronic illness: hypertension, epilepsy, DM, goiter, tumor) before conception? | 1. No 2. Yes |  |
| 303 | | If yes Q.NO 302 which chronic disease? | 1. Hypertension,  2. Epilepsy,  3. Diabetic mellitus  4. Goiter  5. Tumor  6.Other specify____________ |  |
| 304 | | Did you have any illness (fever) one month before or after this pregnancy? | 1. No 2. Yes |  |
| 305 | | Did you admit in health facility in early pregnancy due to sever vomiting? | 1. No 2. Yes |  |
| 306 | | Did you take any drug one month before or after this pregnancy? | 1. No 2. Yes |  |
| 307 | | If yes Q.NO 306 which type of drug/s?  (multiple answer is allowed) | 1. Drug for hypertension  2. Drug for DM  3. Drug for Epilepsy  4. Any other drug |  |
| 308 | | Did you use any traditional herbal medicine one month before or after this pregnancy? | 1. No  2. Yes (specify) __________ |  |
| 309 | | Did you use oral contraceptive before conception? | 1. No 2. Yes |  |
| 310 | | If yes Q.NO 309 for how long? | _________ |  |
| 311 | | Did you have history of NTDs affected pregnancy/live birth baby? | 1. No 2. Yes |  |
| 312 | | Did you have history of congenital anomalies? | 1. No 2. Yes |  |
| 313 | | Was there any close family member had history of NTDs affected pregnancy/live birth baby? | 1. No 2. Yes |  |
| 314 | | Was there any close family member had history of congenital anomalies? | 1. No 2. Yes |  |
| 315 | | Did you take iron/ folic acid/multivitamin before conception? | 1. No 2. Yes |  |
| 316 | | Did you take iron/ folic acid/multivitamin during the current pregnancy? | 1. No 2. Yes |  |
| 317 | | Had history of nutritional deficiency, diagnosed by health professional before conception? | 1. No 2. Yes |  |
| 318 | | If yes Q.N O 319 which deficiency did you diagnosed? | 1.Anemia  2. Energy deficiency  3.Protein deficiency  4. Protein energy deficiency  5.Any other deficiency______ |  |
| Part IV: Environmental factor | | | |  |
| 401 | | Did you take coffee? | 1. No 2. Yes |  |
| 402 | | If yes Q.N 401 how many cups per day (on average)? | _________________ |  |
| 403 | | Did you take tea? | 1. No 2. Yes |  |
| 404 | | If yes Q.N 403 how many cups per day (on average)? | ____________ |  |
| 405 | | Did you drink any alcoholic drink during preconception? | 1. No 2. Yes |  |
| 406 | | Did you smoke cigarette? | 1. No 2. Yes |  |
| 407 | | Was there any cigarette smoker in your home or occupational place? | 1. No 2. Yes |  |
| 408 | | Did you use any pesticides/chemical (insecticides, herbicides or fungicides) at home /work place one month before and after this pregnancy? | 1. No 2. Yes |  |
| 409 | | What is your water source? | 1. Pipe 2. Well  3. Spring 4. Other________ |  |
| 410 | | Did you expose to diagnostic/therapeutic radiation (X-ray) one month before or after this pregnancy? | 1. Yes 2. No |  |
