# Supplementary material for: Risk factors associated to neural tube defects among mothers who gave birth in North Shoa Zone Hospitals, Amhara Region, Ethiopia 2020: Case control study
Source: PLoS One. 2021 Apr 26;16(4):e0250719. doi: 10.1371/journal.pone.0250719 (PMC8075213; doi:10.1371/journal.pone.0250719)
Supplement: S2 File — (DOCX) [file pone.0250719.s002.docx]

የፈቃድ መጠየቂያ ፎርም

**የጥናቱ መረጃ**

ፈቃደኛ ከሆንሸ ለ----------ደቂቃ በፅንስና በህፃናት ላይ ስለሚከሰት የነርቭ ችግረና ችግሩን ሊያመጡ ስለሚችሉ ነገሮች እጠይቅሻለሁ፡፡ ጥናቱም ችግሩን ለመቅረፍ ለሚደረግ ሂደት አስተዋፅኦ አለው፡፡ ፈቃደኛ ከሆንሽ የሚቀጥቀጥሉትን ነጥቦች እነግርሻለሁ፡፡

**የጥናቱ ርዕስ**-በህፃናት ላይ ስለሚከሰቱ የነርቭ ችግሮች እና መንስኤዋቻቸው ነው፡፡

**የጥናቱ ዋና ዓላማ**

የዚህ ጥናት ዋና ዓላማ በሰ/ሸዋ ዞን በሚገኙ ሆስፒታሎች የሚገኙ እናቶች አዲስ በተወለዱ ህፃናትላይ ለሚስተዋለው የነርቭ ችግሮችን መንስኤዋቻቸውን ማጥናት እና የመፍትሄ ሀሳብ ማመላከት ነው፡፡

**በጥናቱ ወቅት ሊከሰቱ ስለሚችሉ ስጋቶች**

ጥናቱ ለችግር የሚያስከትል ወይም የሚያሰጋ ምንም ነገር የለውም፡፡

**ከጥናቱ የሚገኝ ጥቅም ወይም ክፍያ**

በዚህ ጥናት ለሚሳተፉ ተሳታፊዎች ምንም አይነት ቀጥተኛ ጥቅም ወይም ክፍያ የለውም፡፡ ነገር ግን ጥናቱ ከተጠናቀቀ በኋላ በሚገኘው ውጤት እና የመፍትሄ ሀሳብ በተለይ እናቶችን እና ህጻናትን ጤና ለማሻሻል ይረዳል፡፡

**ምስጢራዊነት-**ማንኛውም በዚህ ጥናት የሚሰበሰብ መረጃ ምስጢራዊነቱ የተጠበቀ ነው፡፡ በዚህ መረጃ ስም አይጠቀስም መለያ ኮድ ብቻ ነው የሚሰጠው፡፡

**የጥናቱ የተሳታፊ መብት**

በዚህ ጥናት ለመሳተፍ በመጀመሪያ በተሳታፊ ፍቃድ ላይ የተመሰረተ ነው፡፡በጥናቱ አለመሳተፍ ምንም ጉዳት ወይም ቅጣት የለውም፡፡በመጠይቁ ካሉ ጥያቄዎችም ያልፈለጉትን ያለመመለስ መብት አለዎት፡፡ ባልፈለጉት ሰዓት ከጥናቱ ራስዎን ማግለል ይችላሉ፡፡ በማንኛውም ሰዓት ጥያቄ ካለዎት መጠየቅ ይችላሉ፡፡

ሌሎች ተያያዥ ጥያቄዎችም ካለዎት የዚህ ጥናት ዋና ባለቤት

**አይናለም ጋሻው ኢሜል** [**ayni581@gmail.com**](mailto:ayni581@gmail.com) **እና በስልክ ቁጥር +251-975259688** ደውሎ መጠየቅ ይቻላል፡፡

በመጨረሻም ስለጊዜዎ እናመሰግናለን፡፡ፈቃደኛ ከሆኑ በሚቀጥለው ገፅ ላሉ ጥያቄዎች መልስ ይስጡ፡፡

**የስምምነት ቅፅ**

ከዚህ በላይ የተነበበልሽ ሀሳብ ከገባሽ፣ አሁን በጥናቱ ለመሳተፍ ፍቃደኛ ነሽ?

አዎ ፍቃደኛ ነኝ---------------

ፍቃደኛ አይደለሁም---------------

የተሳታፊ ፊርማ -----------

ቃለ መጠይቅ አድራጊ ስም--------------------------

ጥናት ዋና ባለቤት አይናለም ጋሻው ስልክ ቁጥር +251-975259688

ኢሜል ayni581@gmail.com

የደ/ብርሃን ዩኒቨርሲቲ ኢቲካል ቦርድ ስልክ ቁጥር ________________

መጠይቁ የተደረገበት ቀን________

የመጠይቁ ውጤት 1.ሳያቓርጡ መልስ የሰጡ 2.ያልተገኙ

3.ያቓረጡ 4. በማህል ያቓረጡ.

ፈቃደኛ ካልሆነች ወደ ምትቀጥለው እናት ይሂዱ፡፡

**የሴትዮዋ መለያ ቁጥር ……………………**

- 1. **Annex 4: Amharic Version Questionnaires**

|  | ጥያቄ | መልስ | ምርመራ |
| --- | --- | --- | --- |
|  | በተሳታፊዋ ፅንስ/ልጅ ላይ ከነርቭ ቱቦ መዘጋት ችግር አለ?(እናትየዋን ሳትጠይቅ/ቂ ሙላ/ይ) | 1. አይ 2. አዎ |  |
|  | አዎ ካሉ የተለየው ችግር የቱ ነው?  (እናትየዋን ሳትጠይቅ/ቂ ሙላ/ይ) | 1.አንኢንሴፋሊ  2.ስፓይናልበፊዳ  3.ኢንሴፋሎሴል |  |
| **ክፍል1: ስነ-ህዝብ መረጃ** | | |  |
| ተቁ |  |  |  |
| 101 | ስንት አመትሽ ነው? | _______ ሙሉ ዓመት |  |
| 102 | የጋብቻ ሁኔታሽ ምን ይመስላል? | 1. ያላገባች  2. ያገባች |  |
| 103 | የስራ ሁኔታ/ ምንድነው የምትሰሪው? | 1.የቤትእመቤት  2.ግብርና  3. ነጋዴ  4. የቢሮ ስራ |  |
| 104 | የመኖሪያ አድራሻሽ የት ነው? | 1.ከተማ  2.ገጠር |  |
| 105 | መደበኛ ትምህርት ተምረሻል? | 1. አይ 2. አዎ |  |
| 106 | ጥያቄ ቁ.105 አዎ ካሉ እስከ ስንት? | _______ |  |
| 107 | ባለቤትሽ መደበኛትምህርት ተምሯል? | 1. አይ 2. አዎ |  |
| 108 | ጥያቄ ቁ.107 አዎ ካሉ እስከ ስንት? | _______ |  |
| 109 | በአመት ስንት ኩንታል እህል ያመርታሉ?  (ለገጠር አናቶች) | _______ኩንታል(1000ኪ.ግ) |  |
| 110 | አመታዊ የቤተሰብሽ ገቢ ምን ያህል ነው? (በብር)?(ለከተማ አናቶች) | _______ብር |  |
| **ክፍል2: ከስነተዋልዶ ጤና ጋር የተያያዙ ጥያቄዎች** | | |  |
| 201 | ስንተኛ እርግዝናሽ ነው? | ________እርግዝና |  |
| 202 | ከዚህ በፊት ሞቶ የተወለደ ፅንስ ነበረሽ? | 1. አይ 2. አዎ |  |
| 203 | ከዚህ በፊት ውርጃ አጋጥሞሽ ያውቃል? | 1. አይ 2. አዎ |  |
| 204 | ከዚህ በፊት ባንች ወይም በባለቤትሽ ቤተስብ /የስጋ ዘመድ/ ውሰጥ ውርጃ አጋጥሞ ያውቃል? | 1. አይ 2. አዎ |  |
| 205 | የመጨረሻ ልጅሽን ለስንት ጊዜ አጠባሽ? | ________ወር |  |
| 206 | የእርግዝና ክትትል መቼ ጀመርሽ? | ________ሳምነት |  |
| 207 | ይህን አርግዝና ያረገዝሽው አቅደሽ ነው? | 1. አይ 2. አዎ |  |
| **ክፍል3 ከህክምናጋር በተያያዙ ጥያቄዎች** | | | |
| 301 | ከማርገዝሽ በፊት ለእረግዝና ቅደመ ዝግጅት የምክር አገላግሎት፣ መድሀኒት፣ የአመጋገብ ሁኔታ ለማስተካከል፣ በሽታን ለመከላከል፣ ክትባት፣ አልኮል አንዳትጠጭ ያገኘሽው አገልግሎት አለ? | 1. አይ 2. አዎ |  |
| 302 | ከማርገዝሽ በፊት ተላላፊ ያልሆነ በሽታ እንደ ደም ግፊት፣ ስኳር፣ እንቅረት፣ የሚትል በሽታ /አውድቅ/ ፣እጢ/ካንሰር እንዳለብሽ በሀኪም/ጤና ባለሙያ/ ተነግሮሽ ነበር? | 1. አይ 2. አዎ |  |
| 303 | ጥ.ቁ 302 አዎ ካሉ የተገኘብሽ በሽታ ምን ነበር? | 1.ደም ግፊት  2.የሚጥል በሽታ/አውድቅ/  3.ስኳር  4.እንቅረት  5.እጢ/ካንሰር  6.ሌላይገለፅ____________ |  |
| 304 | ከማርገዝሽ አንድ ወር በፊት እና በአርግዝናሽ አንድ ወር ውስጥትኩሳት/ሌላ ህመም አሞሽ ነበር? | 1. አይ 2. አዎ |  |
| 305 | በእረግዝናሽ መጀመሪያ አካባቢ በከባድ በትውከት /ማስመለስ/ ምክንያት ሀኪም ቤት ተኝተሽ ነበር? | 1. አይ 2. አዎ |  |
| 306 | ከማርገዝሽ አንድ ወር በፊት እና በአርግዝናሽ አንድ ወር ውስጥ የወሰድሽው መድሃኒት ነበር? | 1. አይ 2. አዎ |  |
| 307 | ጥ.ቁ 306 አዎ ካሉ የወሰድሽው መድሃኒት ምን ነበር? | 1. የደምግፊት መድሃኒት  2.የሚጥል በሽታ መድሃኒት  3.የስኳር መድሃኒት  4. ሌላ መድሃኒት |  |
| 308 | ከእርግዝናሽ አንድ ወር በፊት እና በእርግዝናሽ አንድ ወር ውስጥ የተጠቀምሽው የባህል መድሃኒት/ቅጠላቅጠል/ ነበር? | 1. አይ  2. አዎ (ይጠቀስ) ________ |  |
| 309 | ከዚህ በፊት የነርቭ ቱቦ የመዘጋት ችግር በፅንሱ/በልጁ ላይ አጋጠሞሽ ነበር? | 1. አይ 2. አዎ |  |
| 310 | ከዚህ በፊት ሌላ የተፈጥሮ ችግር በፅንሱ/በልጁ ላይ አጋጠሞሽ ነበር?  /ከነርቭ ቱቦ መዘጋት ችግርውጭ/ | 1. አይ 2. አዎ |  |
| 311 | በንች ወይም በባለቤትሽ ቤተሰብ/የስጋ ዘመድ  የነርቭ ቱቦ መዘጋት ችግር ያለበት ፅንስ/ልጅ አጋጠሞ ያውቃል? | 1. አይ 2. አዎ |  |
| 312 | በአንች ወይም በባለቤትሽ ቤተሰብ/የስጋ ዘመድ/ ሌላ የተፈጥሮ ችግር ያለበት ፅንስ/ልጅ አጋጠሞ ያውቃል? /ከነርቭ ቱቦ መዘጋት ችግርውጭ/ | 1. አይ 2. አዎ |  |
| 313 | ይህን እርግዝና ከማርገሽ በፊት አይረን /ፎሌት /ቨይታሚን ወስደሻል? | 1. አይ 2. አዎ |  |
| 314 | በዚህ እርግዝናሽ ወቅት አይረን /ፎሌት/ ቨይታሚን ወስደሻል? | 1. አይ 2. አዎ |  |
| 315 | ከማርገዝሽ በፊት የምግብ እጥረት ችግር እንዳለብሽ በጤና ባለሙያ ተነግሮሽ ነበር? | 1. አይ 2. አዎ |  |
| 316 | ጥያቄ ቁ.315 አዎ ካሉ የተነገረሰሽ የምግብ ችግር ምን ነበር? | 1.ደም ማነስ  2.የሀይልሰጭ ምግብ እጥረት  3. የሰውነት ገንቢ ምግብ እጥረት  4. የሀይል ሰጭና የሰውነት ገንቢ ምግብ እጥረት  5. የሌላ ምግብ እጥረት  ________ |  |
| **ክፍል-4 ከአካባቢ ጋር የተያያዙ ጥያቄዎች** | | | |
| 401 | ቡና ትጠጭአለሽ? | 1. አይ 2. አዎ |  |
| 402 | ጥያቄ ቁ.401 አዎ ካሉ በአማካኝ ስንት ስኒ በቀን? | ________ |  |
| 403 | ሻይ ትጠጭአለሽ? | ­1. አይ 2. አዎ |  |
| 404 | ጥያቄ ቁ.403 አዎ ካሉ በአማካኝ ስንት ብረጭቆ በቀን? | ________ |  |
| 405 | ማንኛውንም የአልኮል መጠጥ ትጠጭያለሽ? | 1. አይ 2. አዎ |  |
| 406 | ሲጋራ ታጨሽያለሽ? | 1. አይ 2. አዎ |  |
| 407 | ቤትሽ ውስጥ ወይም በስራ ቦታ የሚያጨስ ሰው አለ/ለሲጋራ ጭስ ትጋልጫለሽ? | 1. አይ 2. አዎ |  |
| 408 | ከማርገዝሽ አንድ ወር በፊት እና በአርግዝናሽ አንድ ወር ውስጥ የተባይ ማጥፊያ፣የአረም መድሃኒት ወይም ኬሚካል በቤትሽ፣ በአካባቢሽ ትጠቀሚ ነበር? | 1. አይ 2. አዎ |  |
| 409 | ውሀ ከየት ነው የምትጠቀሚው? | 1. ከበቧንቧ  2. ከጉድጓድ/ኩሬ  3. ከምንጭ  4. ከሌላ ________ |  |
| 410 | ከማርገዝሽ አንድ ወር በፊት እና በአርግዝናሽ አንድ ወር ውስጥ የራጅ ህክምና ታዞልሽ ተጠቅመሽ ነበር? | 1. አይ 2. አዎ |  |
